# Learning about climate politics during COP 21: Explaining a diminishing knowledge gap

**DOI:** 10.1177/09636625211068635

**Published:** 2022-01-30

**Authors:** Fenja De Silva-Schmidt, Michael Brüggemann, Imke Hoppe, Dorothee Arlt

**Affiliations:** Universität Hamburg, Germany; University of Bern, Switzerland

**Keywords:** climate change, knowledge acquisition, knowledge gap, media effects, panel survey

## Abstract

A basic understanding of climate politics is necessary for citizens to assess their government’s policies. Media use is supposed to enable learning, while widening knowledge gaps. We analyze whether such a gap opened up in times of intense media coverage during the 2015 climate conference in Paris and explain learning through hierarchical regression analyses, drawing on a 3-month panel survey (*n* = 1121) in Germany. We find a diminishing knowledge gap: people with low previous knowledge catch up on the better informed, but overall knowledge remained low and learning was limited. This suggests a ceiling effect: possibly journalistic media did not provide enough new information for the well-informed. Closing knowledge gaps may also be explained by the media system with public television and regional newspapers reaching broad segments of the population. Higher knowledge was predicted less by media use than by education, concern, and being male.

Climate change is one of the most pressing issues of our time, urgently requiring political action. Without political efforts, the effect of individual engagement, for example, in the form of environmentally conscious consumption choices, is limited. Young activists and scientists agree that political engagement might be one of the most important forms of individual actions against climate change (Hagedorn et al., 2019). A basic knowledge about *climate politics*—defined as knowledge about the main goals, approaches, and actors of politics aiming at preventing or mitigating climate change and its consequences (Delli Carpini and Keeter, 1996; Simonis, 2017)—enables citizens to come up with informed opinions and actions.

Previous research suggests that an important effect of climate-related media coverage is an increase in knowledge about climate change (e.g. Kahlor and Rosenthal, 2009; for Germany: Oschatz et al., 2019; Taddicken, 2013). In turn, general knowledge about the issue positively influences the motivation to act climate-friendly (Bord et al., 2000; Bostrom et al., 2012; Tobler et al., 2012) and supports the willingness and ability to act (Cabecinhas et al., 2008; Kaiser and Fuhrer, 2003; Sterman and Sweeney, 2007), as well as a general pro-environmental stance and a high internal efficacy (Hornsey et al., 2016; Kahan et al., 2012).

While an extensive body of literature confirms a link between media use and climate-related knowledge, current research has begun to identify conditions for it (e.g. Oschatz et al., 2019). To move from correlations to causality, a few experimental studies have been undertaken, but showed only modest effects of media use on climate-related knowledge (Corbett and Durfee, 2004; Moxnes and Saysel, 2009; Sterman and Sweeney, 2007). As an experimental setting hardly represents the everyday exposure to a multitude of often contradictory pieces of information, they suffer from limited external validity. Indeed, qualitative research has shown that learning about climate change is a long-term process (Lörcher, 2018) that usually takes place outside of formal education, for example, induced by media use (Falk et al., 2007; Simcock et al., 2014).

An important but rare approach to explore media effects on learning about climate change in a real-world setting are panel surveys (e.g. Dimitrova et al., 2014). Investigating differential learning over time with longitudinal data has remained a central research desideratum (Gaziano, 2013). One exception is the study of Oschatz et al. (2019), combining a two-wave panel survey with content analysis to investigate individual knowledge about climate change consequences before and after the release of an Intergovernmental Panel on Climate Change (IPCC) report. They show positive effects of prior knowledge and media use, but still only take few potential influencing factors into account.

Our aim is to provide a comprehensive study that integrates most factors that have been shown to explain knowledge and learning from media use in prior studies. Conceptually, we engage with the knowledge gap hypothesis (Tichenor et al., 1970) and its more recent advancements. Sometimes overlooked in past studies, we first of all explore whether there actually is a widening knowledge gap over time.

We apply this framework to the case of learning about *climate politics*, a both relevant and virtually unexamined subject—in contrast to general knowledge about climate change, better explored by past research. We are particularly interested in knowledge about climate policy as an important prerequisite for meaningful political participation. What distinguishes our study is that it explores a learning process in times of high media coverage during an exceptional political event: we conducted a three-wave panel survey in the context of the 2015 UN climate conference (COP 21) that resulted in the Paris Agreement. Thus, the conditions were ideally suited for exploring learning from media content: media attention was guaranteed and highly relevant political decisions on climate politics were taken—yet, we were intrigued to find only modest learning and a diminishing rather than widening knowledge gap.

## Theoretical background and literature review

To explain why some people learn more from media use than others, one of the most common reference points is the knowledge gap hypothesis (Tichenor et al., 1970). In its original version, it posits that people with higher socioeconomic status, measured as formal education, acquire information from media use faster than people with a lower socioeconomic status, resulting in widening knowledge gaps between the two groups. The hypothesis works under the condition of an influx of information, taken as a given if there is media coverage on a topic. Using this hypothesis as a starting point, in the following, we outline how it was expanded by including various predictors that also influence differential learning from the media.

The original hypothesis was much criticized, revised, and adapted (Donohue et al., 1975; Eveland and Scheufele, 2000; Liu and Eveland, 2005), but all revisions maintain the basic assumption that learning processes are influenced by *sociodemographic factors*, that is, *formal education*. In many studies, formal education is used as a proxy for previous knowledge, since it is easy to ascertain in a survey. Indeed, many empirical studies have shown a relevant positive correlation between formal education and political and scientific knowledge levels (Aarts et al., 2012; Lind and Boomgaarden, 2019; Miller, 2010), also in the field of climate communication (Lee et al., 2015; Tobler et al., 2012). However, panel studies only partly support a direct relation between formal education and learning: Oschatz (2018) shows education to have a positive effect only on learning about the consequences of climate change, but not on its causes and counter-measures. Milfont (2012) did not find direct effects of education on learning about climate change.

Another important sociodemographic factor, not included in the original hypothesis but identified in empirical research, is *gender*: In most studies on political and scientific knowledge, males know more than females (Delli Carpini and Keeter, 1996; Dolan, 2011; Lizotte and Sidman, 2009; Price, 1999; Takahashi and Tandoc, 2016). However, the picture is inconclusive for the topic of climate politics. Studies show that females not only possess more accurate knowledge (McCright, 2010), but also learn more about climate change consequences (Oschatz, 2018) than males—possibly related to the fact that climate change denial is more common among men than women (Whitmarsh, 2011), and that women feel more concerned about climate change (McCright, 2010). Thus, it is unclear in which direction possible gender effects for the topic of climate politics may work. Age and income do not seem to have a systematic influence on learning about political and scientific topics (Eveland and Scheufele, 2000; Oschatz, 2018).

Advancements of the original knowledge gap hypothesis have stressed that besides general and constant sociodemographic factors, situational factors on the individual’s side also matter (Donohue et al., 1975). First, Ettema and Kline (1977) included *motivational factors*, such as the perceived benefit of an information, into the model of learning from media. Contrary to the previous, negative perspective on the knowledge *deficit*, they neutrally interpret it as a knowledge “difference”, and posit that *personal interest in a topic* is an important prerequisite to acquire knowledge (an assumption empirically supported especially for health topics, Ettema et al., 1983). Studies have shown the importance of personal interest especially for free-choice learning, as opposed to formal learning within the school system (Falk et al., 2007), which is mostly the case for learning about climate politics. A high personal interest in a topic also makes a high elaboration of information more likely, fostering knowledge acquisition (Eveland, 2001; Eveland and Scheufele, 2000; Eveland et al., 2003; Petty and Cacioppo, 1986). Apart from personal relevance, another important motivational factor for the topic of climate politics might be *climate skepticism*, or the acceptance of the scientific consensus about anthropogenic global warming as a “gateway belief” (Van der Linden et al., 2019). People who deny the existence of (anthropogenic) climate change are quite resistant to learning about the subject due to motivated reasoning—they lack the motivation to acquire accurate factual information (Hart et al., 2015; Whitmarsh, 2011).

Second, individuals must not only be motivated to process the information they receive, they also need the ability to do so. Tichenor et al. (1970) mentioned that *cognitive factors*, such as an extensive *subject-specific previous knowledge* and a higher *information literacy*, could be the underlying factors explaining knowledge gaps between better and less educated individuals. Especially when information is complex, background knowledge is needed to process and memorize it correctly. For political knowledge, Price and Zaller (1993: 157) have shown “that citizens’ likelihood of learning about current news events is best predicted by pre-existing knowledge of political affairs.” Likewise, the panel survey of Oschatz et al. (2019) shows that prior knowledge is one of the strongest positive predictors of learning about climate change. The study was able to prove a weak learning effect about climate change consequences from news use after the release of an IPCC report. A high information literacy, meaning the ability to understand new pieces of information and to integrate them into one’s own knowledge, also makes learning from media more likely (e.g. Bird et al., 2011).

Apart from the aforementioned factors, research on knowledge gaps suggests that different *types of media use* vary in their influence on learning processes. *Newspaper use* seems to foster learning in general, but gives an advantage to people with a higher formal education (Tichenor et al., 1970). In contrast, people with all education levels can learn something from *television use*, especially those with a lower subject-specific knowledge—it thus seems to serve as a knowledge leveler, closing knowledge gaps (Eveland and Scheufele, 2000; Kwak, 1999; Tichenor et al., 1970). For climate-related knowledge, Zhao et al. (2014) showed in a 1-year field experiment that watching television news lead to learning effects. This finding is supported by the panel survey of Taddicken (2013), where television was the only type of media with a slightly positive effect on individual knowledge levels. However, studies have pointed out that only high-quality public television news have such a beneficial effect on knowledge, while private TV channels often have no positive or even negative effects (e.g. Aalberg and Curran, 2012a)—it thus seems important to differentiate these different subtypes of media when measuring media use.

Since retrieving reliable information from the *Internet* can be a challenging task, mainly people with a higher education learn from Internet use, adding to the so-called “digital divide”; a gap in knowledge between people with access to the Internet and sufficient skills on the one side, and those with no access on the other (Van Deursen and van Dijk, 2011). However, in many studies, the correlation between online media use and political knowledge was weak or not significant (Dimitrova et al., 2014; Ho and Yang, 2018; Maurer and Oschatz, 2016). As for the other media, learning effects likely depend on the type of outlet and sources used on the web rather than on Internet use per se.

For climate-related knowledge, Stamm et al. (2000) found that *interpersonal communication* was also positively associated with understanding climate change, as it serves both as a source of information and as a forum of elaboration, helping to understand and turn information into knowledge.

## Research questions and hypotheses

We pose two research questions, one on the collective and the other on the individual level. On the collective level, we first want to know: *How does public knowledge about climate politics evolve in times of high media coverage—does the gap in knowledge about climate politics widen, stagnate or diminish*? The original hypothesis expects a widening knowledge gap “as the infusion of mass media information into a social system increases” (Tichenor et al., 1970: 159). However, subsequent research has identified a number of limiting conditions to this hypothesis (Donohue et al., 1975). Furthermore, different ceiling effects induced by the information sources or the audience (Ettema and Kline, 1977) can prevent a growing knowledge gap. It is unclear how the public’s knowledge of climate politics—a global, sometimes contested and complex topic—evolves in times of intense media coverage during a climate summit.

The second question focuses on explaining individual levels of knowledge, situating media and communication effects among other factors: *Which factors explain differences in learning about climate politics*?

First, the sociodemographic variables gender and education are expected to have an effect. Since the literature has identified diametrically opposed gender effects for political topics and the topic of climate change, the effect of gender on learning about climate politics remains unclear, thus we propose addressing it empirically in form of a non-directional hypothesis:

*H1*. Men and women differ in terms of the amount of knowledge that they learn.
The second hypothesis also refers to a sociodemographic factor, based on the knowledge gap hypothesis:
*H2*. The more formal education individuals have, the more they learn about climate politics.
Motivational factors are expected to have an influence:
*H3*. The more personal importance individuals ascribe to climate change, the more they learn about climate politics.
*H4*. The stronger individuals deny the scientific consensus concerning anthropogenic climate change, the less they learn.
Subsequently, the cognitive resources available to process new information are expected to have a positive effect:
*H5*. The higher individuals’ ability to understand information about climate change in the media sources they use, the more they learn about climate politics.
*H6*. The more subject-specific previous knowledge individuals have, the more they learn about climate politics.
Finally, the following types of media use are expected to have a positive effect:
*H7*. The more informational newspaper an individual uses, the more they learn about climate politics.
*H8*. The more an individual uses public television, the more they learn about climate politics.
*H9*. The more an individual talks about climate politics with others, the more they learn about the topic.

## Methods

To analyze our research question and test the hypotheses, necessary preconditions are identifying a learning process on the individual level and tracking the evolution of knowledge levels collectively. We conducted a panel survey during the course of the 2015 climate conference in Paris (COP 21). This event was chosen because the UN climate conferences are main triggers of media coverage on climate change (Schmidt et al., 2013), and serve as global media events that focus the public’s attention on the topic (Brüggemann et al., 2017; Wozniak et al., 2021). If it was possible to identify and analyze learning effects, it would be most probable during this time. The knowledge gap hypothesis also refers to situations with an influx of new information into the social system—which is given during COP 21, resulting in the Paris Agreement.

While most previous studies focus on the US context, our survey was conducted in Germany. Here, climate change is seen as an important and barely controversial topic (De Silva-Schmidt and Brüggemann, 2019; Schäfer, 2016), and media coverage and audience engagement with the climate summit were likely to be high, possibly enhancing learning effects.

### Design of the study

#### Time frame

The panel survey took part 2 weeks before, during, and 4 weeks after the UN climate conference 2015 (COP 21). For the following analyses, we only use data from the first and the last wave (conducted in November 2015 and January 2016) to avoid conclusions based on short-term changes during the conference period, since we are interested in learning as a long-term effect.

#### Sample

The external panel provider respondi (certified according to ISO 26362) recruited the respondents via an online access panel with 100,000 respondents in Germany. First, participants from the panel were randomly invited to take part in the survey. Second, a quota regarding age and gender, federal state, and formal education was applied to the sample for the first survey wave to represent the distribution of these variables within the German population aged 18–69. The final sample after the third wave comprised *n* = 1121 participants (wave 1: *n* = 2098).

### Measures

#### Learning about climate politics

In contrast to much of the literature, we focus on learning about climate politics. This is why the knowledge scale was only partly based on previous surveys, that have not covered climate *politics*, but concentrated on knowledge of causes, consequences, and individual counter-measures of climate change (Reynolds et al., 2010; Shi et al., 2016; Sundblad et al., 2009; Tobler et al., 2012). Our items (see Table a in the supplemental material) cover the three main dimensions of climate politics as defined by Simonis (2017), in particular knowledge about its main goals (e.g. the 2 degree target), approaches (e.g. mitigation and adaptation), and actors (e.g. CO_2_ emissions of states). Apart from two items (concerning the Kyoto Protocol and emissions trading), which were modified from a study on political knowledge (Trepte et al., 2017), all items were newly developed and can thus be seen as explorative.

The items cover different aspects that are, arguably, highly relevant to gain a basic understanding of climate politics—enabling citizens to meaningfully participate in climate debates and to engage in the democratic process. To judge whether, for example, the German government engages sufficiently in climate protection, it seems vital to know how Germany’s per capita emissions fare in comparison with a country like India. To evaluate whether climate actions are efficient, it is highly important to know that global emissions have been rising rather than shrinking since international efforts started. If basic terms like emission trading or mitigation are unknown, it will be hard to follow public debates about climate politics.

The questions vary in their level of difficulty, as shown by a qualitative pre-test with graduate students. In addition, the questions were validated by an independent expert from the Climate Service Center Germany of the Helmholtz-Zentrum Geesthacht. Our knowledge scale includes both basic background knowledge (e.g. if CO_2_ emissions have been reduced so far) and knowledge that is more closely related to the specific summit (e.g. asking for the key objective of COP 21). The seven items are multiple-choice questions with four alternative answers plus the option to respond with “don’t know.” For the analyses presented in this article, correct answers were coded as 1, while incorrect and “don’t know” answers were coded as 0. The learning effect is operationalized as the difference between the first wave and the last wave of the survey, with knowledge in wave 3 representing the dependent variable of the regression.

#### Influencing factors

Gender (H1) was captured as a dichotomous variable (47.4% female); for education (H2), we used a 5-point Likert-type scale from 1 “no graduation (yet)” to 5 “university diploma” (*M* = 3.1, *SD* = 1.09).

The motivation to process information about climate change was measured by taking personal relevance of the topic (H3) as a proxy (Arlt et al., 2011). The respondents indicated how important climate change was for them personally on a 5-point Likert-type scale ranging from 1, “not at all important,” to 5, “very important” (*M* = 3.84, *SD* = 1.01; see also Frick et al., 2004; Malka et al., 2009). Climate change denial (H4) was captured as a mean index of four items on a 5-point scale ranging from “do not agree” (1) to “fully agree” (5), higher values indicating more denial (*M* = 2.21, *SD* = 0.85; Brüggemann and Engesser, 2014). As information literacy concerning climate change (H5) is often measured as the subjective understanding of sources, participants indicated if they found the information on climate change in the media used by themselves easy to understand on a 5-point Likert-type scale from 1, “do not agree at all,” to 5, “totally agree” (*M* = 3.66, *SD* = 0.92). Topic-specific previous knowledge (H6) sums up the correct answers in the first wave of the survey (*M* = 2.42, *SD* = 1.59).

Media use (H7 and H9) was included in form of habitual media use, since we expect an effect on knowledge from the media use aggregated over time rather than from single occasions. We measured the habitual use of media sources with a 7-point Likert-type scale ranging from 1 (“never”) to 7 (“several times daily”). For all types of media, examples were provided. The sources included were public television (*M* = 4.84, *SD* = 1.84), commercial television (*M* = 4.14, *SD* = 1.91), national print newspaper (*M* = 2.15, *SD* = 1.51), weekly newspaper or magazine (*M* = 2.12, *SD* = 1.28), regional print newspaper (*M* = 3.63, *SD* = 2.00), tabloid newspaper “BILD-Zeitung” (*M* = 2.00, *SD* = 1.56), the most widely used online newspapers spiegel.de (*M* = 2.25, *SD* = 1.61) and bild.de (*M* = 2.09, *SD* = 1.69), other online newspaper (*M* = 2.27, *SD* = 1.66) and interpersonal discussions (*M* = 4.10, *SD* = 1.45).

#### Control variables

The models used in the analysis included the control variables age (*M* = 47.89, *SD* = 13.00) and monthly income (Mdn = 2000–2999€).^1^

## Results

We found a rather low initial level of public knowledge about climate politics (*M T*1 = 2.42 correct answers on the index of seven items; for frequencies, see Table b in the supplemental material). Comparing the group of people with low previous knowledge (0–2 correct answers) to the group of people with higher than average knowledge in *T*1 (3–7 correct answers), we found that the former had a significantly lower formal education and less income (see Table c in the supplemental material)—a typical set of factors accompanying knowledge gaps (Table 1). Over the course of the climate conference, there was a significant, but small collective learning effect: People were able to give 0.22 correct answers more in the mean than before (*t*(1120) = 5.42, *p* < .001).

**Table 1.** Mean differences between wave 1 and wave 3, high/low knowledge groups separated.

Group	*M* (*SD*) *T*1	*M* (*SD*) *T*3	Δ	*n*
High previous knowledge	3.80 (0.94)	3.60 (1.50)	−0.20	543
Low previous knowledge	1.14 (0.82)	1.76 (1.37)	0.62	578
Δ between groups	2.66***	1.84***	0.82	1121
Total	2.42 (1.59)	2.65 (1.70)	0.22	1121

*** = *p* < .001; *SD*: standard deviation.

To address our first research question and find out whether the gap in knowledge between the two groups increased, decreased, or stagnated, we analyzed the mean difference and ran an additional *t* test (Table 1).

In wave 1, the mean difference between both groups was 2.66 correct answers. In wave 2, the difference was only 1.84 correct answers, since the “low previous knowledge” group had improved their score, while the “high knowledge” group had given less correct answers in the mean. To test whether this difference between the two groups was significant, a new variable for the learning effect was calculated (a sum index of the difference between the first and second waves per item), which indicates whether a person performed better, equally well, or worse after the climate summit than before. Subsequently, the learning effect was compared between the two groups by an unpaired *t*-test. We found that the knowledge gap between better and less informed citizens diminished significantly by 0.8 correct answers (*t*(1061.8) = 10.28, *p* < .001), contrary to the assumption of the knowledge gap hypothesis.

To explain these differences in learning, in the following, we present the results of our hierarchical regression analysis. The final model fulfills all necessary preconditions for a multiple regression and was able to statistically significantly predict learning, *F*(18, 911) = 47.348, *p* < .001. The *R*² for the final model was .483 (adjusted *R*² = .473), thus explaining roughly half of the variance, indicative for a high goodness-of-fit according to Cohen (1992). Table 2 shows the results in detail.

**Table 2.** Results of hierarchical regression analyses.

	**Model 1**			**Model 2**			**Model 3**		
	*B*	*SE*	β	*B*	*SE*	β	*B*	*SE*	β
***General individual factors***									
Gender (1 = female)	**−0.74**	**0.10**	−0.222***	**−0.44**	**0.08**	−0.133***	**−0.42**	**0.08**	−0.126***
Education	**0.53**	**0.05**	0.339***	**0.28**	**0.04**	0.179***	**0.23**	**0.05**	0.148***
Age	**0.01**	**0.00**	**0.107** ***	0.01	0.00	0.050***	0.00	0.00	0.027***
Income	**0.10**	**0.04**	**0.081** ***	0.04	0.03	0.031***	0.01	0.03	0.009***
***Topic-specific individual factors***									
Personal importance				**0.16**	**0.05**	**0.097** ***	**0.15**	**0.05**	**0.088** ***
Climate change skepticism				0.04	0.06	0.022***	0.06	0.06	0.029***
Information literacy				**0.20**	**0.04**	0.113***	**0.16**	**0.04**	0.092***
Knowledge T1				**0.57**	**0.03**	0.528***	**0.54**	**0.03**	0.503***
***Media use***									
Public TV							0.05	0.03	058***
Commercial TV							**−0.05**	**0.02**	**−0.060** ***
National print newspaper							−0.01	0.04	−0.013***
Weekly newspaper or magazine							0.01	0.05	0.010***
Regional print newspaper							**0.05**	**0.02**	**0.059** ***
Tabloid newspaper (BILD)							−0.03	0.03	−0.032***
spiegel.de							**0.07**	**0.04**	**0.069** ***
bild.de							−0.01	0.03	−0.006***
Other online newspaper							0.00	0.03	0.001***
Interpersonal discussions							0.01	0.03	0.012***
**Adjusted *R*²**	0.157			0.464			0.473		
***F* (*df*1, *df*2)**	44.142*** (4, 925)			101.595*** (8, 921)			47.348*** (18, 911)		
**∆*F* (*df*1, *df*2)**	n.a.			133.715*** (4, 921)			2.567** (10, 911)		

Significant predictors are printed in bold.

*** *p* < .001; **p < .01; *p < .05.

We see that the sociodemographic variables gender and education have a significant effect in all models. A gender difference is obvious (H1): Males have learnt more about climate politics by the end of the climate summit. There is also a positive effect of formal education (supporting H2), consistent with the knowledge gap hypothesis. However, the sociodemographic variables only explain less than 2% of the variance.

Motivational and cognitive factors prove most important to explain higher knowledge levels after the climate summit. A higher personal importance of climate change correlates with a higher knowledge on the topic, as expected by Hypothesis 3. However, climate change skepticism has no effect; thus, Hypothesis 4 is rejected. Higher information literacy concerning climate change coverage is also associated with higher knowledge levels (H5 is supported). Prior knowledge about climate politics positively correlates with the final knowledge level. Yet this does *not* support our sixth hypothesis that higher knowledge will lead to more learning, as we will argue below when discussing this result in light of the prior result of a diminishing knowledge gap. The set of topic-specific individual factors raises the explained variance of the model by 30%.

In the final regression model, we additionally included media use. Reading regional print newspapers and the online newspaper spiegel.de has a small positive effect on knowledge (H7 is only partially supported, as national quality newspapers and magazines have no effect). While public television has no significant effect (H8 is rejected), commercial television even seems to interfere with learning. No other sources show a significant effect (H9 concerning interpersonal discussion is also rejected). All media effects are small and significant only on a low level—all types of media use combined only add less than 2% of explained variance to the model.

## Discussion

To sum up the analysis and findings: We conducted a regression analysis on panel survey data to find out how public knowledge about climate politics evolves in times of high media coverage, and which factors influence learning about climate politics. Based on the knowledge gap hypothesis and its advancements, we included sociodemographic, cognitive, and motivational factors as well as a differentiated measurement of media use in our analysis. Prior knowledge was the most important factor to predict knowledge after the COP 21. This is not surprising as the overall learning effect was small and knowledge levels remained mostly stable. Learning is positively influenced by higher education and (male) gender. Higher motivation to process climate information and self-assessed information literacy generated a moderate positive effect. The use of regional newspapers and spiegel.de had a minor positive effect, while private television had a minor negative effect.

These findings advance our understanding of learning from media content in three ways by advancing and challenging some assumptions related to the knowledge gap hypothesis: We find that factors explaining learning and the evolution of knowledge gaps are far more dependent on the sociocultural context than prior discussions suggest—both the media system and issue-specific national opinion cultures play a role.

Our first and most important finding challenges the assumption of a widening knowledge gap during a period of intense media coverage. With regards to the distribution of knowledge about climate policy among the German public, there is no widening gap. Instead, the gap between those who are ignorant about a number of substantial aspects of climate policy and those who are better informed shrinks. Media coverage may thus have served as a leveler of knowledge—albeit on a fairly low level of knowledge before and after the summit and fairly small overall learning effects. Being one of few studies that have actually tested the evolution of knowledge over time (e.g. Milfont, 2012; Oschatz et al., 2019; Wanta and Elliott, 1995), and the only study to explore knowledge about climate politics in this way, we find no evidence for a *widening* knowledge gap as a media effect.

One explanation could be that knowledge gap research has (a) neglected the contextual factor of the media system and (b) often not measured media effects in a differentiated way, distinguishing different types of, for example, television or online sources. In our data, the regional press and spiegel.de have fostered knowledge gains, while private television correlates negatively. Both positive factors reach very broad audiences in Germany, so that they were able to diminish the knowledge gap. For example, in the United States, public television is very weak, the regional press and elite outlets are less widely read today, and private television (in our study, a negative factor) is very strong, leading to different effects on knowledge gaps. Thus, it is plausible that media coverage, representing the total output of a media system, may have different effects in countries with different media systems.

Second, the knowledge gap hypothesis is to some degree not a media effect hypothesis, but rather describes an “education gap” effect, since education predicts learning about climate politics far better than media use—confirming the findings of most other studies on the important influence of education.

Third, we find that national issue cultures influence the power of certain explanatory factors. Surprisingly, political orientation and climate change denial were not relevant to explain learning about climate politics. This is interesting because in many studies analyzing knowledge about and attitudes toward climate change, these variables are significant predictors (e.g. Hamilton et al., 2015; Hart et al., 2015; McCright and Dunlap, 2011). However, most of these studies were conducted in the United States. It seems that in this regard, the US population cannot serve as a representative sample for other Western countries. Climate change is a much more controversial issue there than in, for example, the German context. In addition, the political system of the United States with only two major parties is special, which further reduces the generalizability of results concerning political knowledge stemming from the United States (Aalberg and Curran, 2012b: 4). This shows that for the topic of climate change, a concentration on studies from the US context is problematic and results should not be generalized to other Western democracies. Also, learning processes are embedded in a sociocultural context.

In line with prior research (e.g. Ettema et al., 1983), those who were more motivated to consume news about the topic because of a higher perceived personal relevance learnt more. This is a relevant finding for practical climate communication. The other factors influencing knowledge gaps are hard to change (level of education, media systems, and issue cultures)—but it is possible to communicate the personal relevance of, for example, climate politics more effectively.

Finally, it is important to discuss our descriptive finding of an overall small learning effect. Knowledge levels remained mostly stable, and well-informed people learnt even less than people with low prior knowledge. To understand why people with a high previous knowledge on climate politics did not learn more, ceiling effects come into play. First, a methodological ceiling effect comes to mind, but does not seem plausible: Only 0.4% of the participants (five persons out of 1121) were able to answer all questions correctly in the first wave of the survey (see Table b in the supplemental material)—of course, those persons with a “perfect” previous knowledge score were not able to learn in the course of our study, but their share is extremely minor.

Second, another type of ceiling effect that may have played a role relates to motivational factors: we see in the regression analysis that motivation is fairly important to explain learning. Maybe participants with a moderate to high knowledge level felt an “internal saturation” and perceived themselves as knowledgeable enough. Without an interest in learning more, they did not acquire new information (called an “audience-imposed ceiling” by Ettema and Kline, 1977: 198). However, we would argue that the feeling of being informed was an illusion as most people failed to correctly respond to important questions concerning basic climate policy-making.

This issue could also be tied to miscalibration between perceived competence and objective knowledge (Fischer et al., 2019, 2018)—people with a basic knowledge might have felt they possessed sufficient knowledge and thus lacked motivation to learn more. This impression of being informed after having acquired a basic knowledge has been called the “illusion of knowing” by Park (2001), which can possibly have negative effects on the motivation to learn more. This assumption cannot be verified because we did not directly measure *perceived* knowledge or people’s subjective need for information in our study. Future studies on learning effects should therefore include these variables.

However, a lack of motivation to acquire new information does not seem very likely, since a study on the German audience’s reception of and attitudes toward COP 21 coverage rather shows the public’s dissatisfaction with the reporting, especially due to missing background information (De Silva-Schmidt and Brüggemann, 2019).

Thus, we assume that methodological and motivational ceilings are not sufficient to explain our findings. We propose a third ceiling effect concerning the influx of information: if media coverage did not offer the kind of information needed to respond to our knowledge questions—at least not the type of media used by the majority of the audience, which was mostly TV—only people with a low level of knowledge could learn new facts from the coverage, while those with a basic knowledge on some questions could not learn much on the other items. In the literature, this has been called a “source-imposed ceiling” by Ettema and Kline, 1977: 198; our results are similar to Wanta and Elliott’s (1995) panel study on HIV knowledge. Although we expect there were in-depth articles or broadcasts provided, they apparently were rare or did not reach broad audiences. This explanation seems plausible as it would also explain why the effect of media use related to the climate summit is so small and barely significant.

However, only a content analysis covering our knowledge items could confirm this assumption. Existing content analyses of COP 21 coverage already show that most of the media reported heavily in the beginning of the summit and much less on the developments further into the conference (Painter, 2016; Schäfer et al., 2016), which might have been detrimental to continuous learning. It seems the media reporting focused on a narrow range of information that was mostly event related.

Concerning mass media use, the knowledge gap hypothesis seems to be subject to strong media ceiling effects when looking at certain media types with broad audiences, but not much deeper information to offer, such as tabloids and many private television stations. A limited influx of information can only contribute to closing knowledge gaps between uninformed persons and people with basic knowledge. A motivational ceiling effect may additionally have occurred when people felt well-informed after having acquired some pieces of information.

## Conclusion and outlook

Our study may thus serve as a substantive complement to existing studies on the knowledge gap hypothesis. We can confirm the hypothesis’ main assumption of more educated people having learnt more in the end, but we also see that the gap between people with higher and lower knowledge levels actually decreases, thus putting into question the general assumption of widening knowledge gaps. After this time of intense media coverage, knowledge seems to become more evenly distributed, as the group of people with lower knowledge levels at the start learnt more than people with higher previous knowledge.

In our study, learning is mostly explained by motivation and sociodemographic factors, while only some types of media (regional newspaper and the leading online newspaper) have a small positive effect. Prior knowledge positively predicts knowledge levels after the summit—however, the difference in knowledge between well-informed and less informed people diminishes in a media environment that also provides some basic knowledge to the less educated publics.

This shows that potential knowledge gap effects can be overshadowed by a media system leading to leveling of knowledge and ceiling effects due to limited provision of in-depth information in the most commonly used media outlets. However, this assumption of a media ceiling effect needs to be confirmed with a content analysis. A combination of a panel survey with a content analysis would be especially insightful.

We measured factual knowledge with close-ended multiple-choice items. Within a panel survey like ours, other types of knowledge, such as structural knowledge, are difficult to include, but would be relevant to analyze learning in its entirety—other studies could go deeper in this regard. Furthermore, our items are a normative selection of facts we deem relevant to understand climate politics. Yet, we do not claim to have covered all facets of climate politics. Furthermore, there is no objective standard as to which aspects are most important to know for informed citizens (see, e.g. Graber, 2001). While we can justify our choice of items, it must remain open to debate whether any important aspects are missing. Broadening our list of knowledge items is certainly desirable for future studies.

Although we used a panel survey design, the timeframe for learning was rather short, covering only 3 months. Thus, future studies could look into learning effects over a much longer period of time. We still expect media to contribute a relevant effect to learning in the long term, in a dynamic process combined with other factors, and maybe more on issues not covered by our items. The effect of media use seems to be weak in the case of the relatively short-term event-specific learning. Qualitative studies analyzing a concrete learning process could also contribute valuable insights into how individuals make sense of the information they receive in their media repertoires and thus acquire new knowledge.

Our results show that the learning process from media use cannot be explained by disregarding the information environment provided by media systems, issue-specific political discourse cultures and differences between types of media. Going beyond the limits of this study, by combining long-term panel studies with qualitative analyses, is worth further research.

## Supplemental Material

sj-docx-1-pus-10.1177_09636625211068635 – Supplemental material for Learning about climate politics during COP 21: Explaining a diminishing knowledge gap Click here for additional data file. Supplemental material, sj-docx-1-pus-10.1177_09636625211068635 for Learning about climate politics during COP 21: Explaining a diminishing knowledge gap by Fenja De Silva-Schmidt, Michael Brüggemann, Imke Hoppe and Dorothee Arlt in Public Understanding of Science

## Notes

1. Political orientation (7-point scale ranging from “extreme left” (1) to “extreme right” (7), *M* = 3.79, *SD* = 1.03) was excluded for two reasons: By inclusion of the variable, more than 100 cases would have been omitted due to missing values; furthermore, there was no correlation between political orientation and learning effects.
