# Supplementary material for: Learning about climate politics during COP 21: Explaining a diminishing knowledge gap
Source: Public Underst Sci. 2022 Jan 30;31(5):617–33. doi: 10.1177/09636625211068635 (PMC9131408; doi:10.1177/09636625211068635)
Supplement: sj-docx-1-pus-10.1177_09636625211068635 – Supplemental material for Learning about climate politics during COP 21: Explaining a diminishing knowledge gap [file sj-docx-1-pus-10.1177_09636625211068635.docx]

**Learning about climate politics during COP 21**

**Explaining a diminishing knowledge gap**

**- Supplemental Material –**

Fenja De Silva-Schmidt^1^, Michael Brüggemann^1^, Imke Hoppe^1,2^, Dorothee Arlt^3^

^1^Universität Hamburg, Institute for Journalism and Communication Studies

^2^German Aerospace Center, Institute of Systems Engineering for Future Mobility

^3^University of Bern, Institute for Communication and Media Studies

Corresponding author: Fenja De Silva-Schmidt, fenja.ds.schmidt@gmail.com.

This article is part of the project “Down to Earth (D2E)” at the University of Hamburg, Germany. For more information, see:

https://www.wiso.uni-hamburg.de/en/fachbereich-sowi/professuren/brueggemann/forschung/forschungsprojekte/projekt4-downtoearth-projekt.html

**Table of Contents**

[*Table a. Wording of knowledge questions and answering options. 2*](#_Toc89336528)

[*Table b. Frequency of correctly answered knowledge items (wave 1 and 3). 3*](#_Toc89336529)

[*Table c. t test between groups of high and low previous knowledge regarding SES. 3*](#_Toc89336530)

[*Table d. Sample demographics. 3*](#_Toc89336531)

[*Questionary for independent variables. 4*](#_Toc89336532)

[*1. Personal importance of the topic 4*](#_Toc89336533)

[*2. Climate change skepticism 4*](#_Toc89336534)

[*3. Information literacy 4*](#_Toc89336535)

[*4. Media use 5*](#_Toc89336536)

[*Table e. Descriptive results of independent variables in the first wave 6*](#_Toc89336537)

## *Table a. Wording of knowledge questions and answering options.*

| **Nr.** | **Question wording** |
| --- | --- |
| 1 | To protect the climate, many countries have signed an agreement which limits the greenhouse gas emission levels (e.g. CO_2_). What is the name of this agreement?  - CO_2_-Stop Agreement  - Tokyo Resolution  - Kyoto Protocol*  - Agenda G13  - Don’t know |
| 2 | International climate policy is aimed at decreasing greenhouse gas emissions. Have carbon dioxide (CO_2_) emissions actually been reduced?  - Compared to 1990, the worldwide CO_2_ emissions are currently about 50% higher.*  - Since 1990, the worldwide CO_2_ emissions have decreased by about one third.  - Compared to 1990, the worldwide CO_2_ emissions have more or less remained the same.  - Following the ban of CFCs, the worldwide CO_2_ emissions have practically dropped to zero.  - Don’t know |
| 3 | What is the name of an economic instrument with which politics currently attempts to achieve climate protection goals?  - Emissions trading*  - Social welfare  - Financial compensation  - Scrapping premium  - Don’t know |
| 4 | What does the term mitigation mean? Measures that…  - …stop or slow down the greenhouse-related changes of climate.*  - …slow down industrialization in developing countries.  - …cut back the industry in industrial nations.  - …reduce the governments' national influence on climate protection.  - Don’t know |
| 5 | What is the key objective of this year's climate conference in Paris? The goal is...  - … to pass a new binding climate protection agreement under international law.*  - … to draw up new scientific progress reports on the consequences of climate change.  - … to prepare climate protection measures specifically for developing countries.  - … to commit emerging countries to climate protection goals.  - Don’t know |
| 6 | The so-called two degree objective describes the goal…  - ...to limit the increase in the worldwide average temperature to less than two degrees Celsius compared with the level prior to the beginning of the industrialization.*  - …to limit the increase in the worldwide average temperature by no more than two degrees Celsius per year compared with the level prior to World War I.  - …to limit the increase in the worldwide average temperature to less than two degrees Celsius compared with the level prior to the foundation of the UN.  - …to limit the increase in the worldwide average temperature to less than two degrees Celsius compared with the level prior to the first climate conference in 1995. |
| 7 | The CO_2_ emissions are one of the main causes of climate change. Which of the following countries has the lowest level of CO_2_ emissions per capita?  - Germany  - USA  - India*  - Qatar  - Don’t know |

## *Table b. Frequency of correctly answered knowledge items (wave 1 and 3).*

|  | **wave 1** | | **wave 3** | |
| --- | --- | --- | --- | --- |
| **number of correct answers** | **frequency** | **percent** | **frequency** | **percent** |
| 0 | 162 | 14.5 | 137 | 12.2 |
| 1 | 175 | 15.6 | 179 | 16.0 |
| 2 | 241 | 21.5 | 226 | 20.2 |
| 3 | 263 | 23.5 | 211 | 18.8 |
| 4 | 164 | 14.6 | 197 | 17.6 |
| 5 | 85 | 7.6 | 120 | 10.7 |
| 6 | 26 | 2.3 | 40 | 3.6 |
| 7 | 5 | 0.4 | 11 | 1.0 |
| total | 1121 | 100 | 1121 | 100 |

## *Table c. t test between groups of high and low previous knowledge regarding SES.*

| **Variable** | **M (high knowledge)** | **M (low knowledge)** | **Δ** | **n** | p | **T** | **df** |
| --- | --- | --- | --- | --- | --- | --- | --- |
| gender  (0 = male) | 0.40 | 0.55 | 0.15 | 1121 | .000 | 5.109 | 1116.76 |
| formal education | 3.29 | 2.91 | 0.38 | 1121 | .000 | 5.906 | 1102.38 |
| income | 3.02 | 2.70 | 0.32 | 982 | .000 | 3.745 | 956.96 |

## *Table d. Sample demographics.*

| **Wave** | **Time frame** | **Respondents** | **Sociodemographic indicators** |
| --- | --- | --- | --- |
| 1 | November 10, 2015 - November 18, 2015 | 2098 | 1031 male, 1067 female;  age *M* = 44.16 years, *SD* = 13.87 |
| 2 | December 6, 2015 - December 10, 2015 | 1477 | 760 male, 717 female;  age *M* = 46.2 years, *SD* = 13.42 |
| 3 | January 11, 2016 –  January 18, 2016 | 1121 | 590 male, 531 female;  age *M* = 47.89 years, *SD* = 13 |

## *Questionary for independent variables.*

### *Personal importance of the topic*

**How important to you are the problems due to climate change?**

1. not important at all
2. not very important
3. somewhat important
4. rather important
5. very important

### *Climate change skepticism*

**Next we would like to ask you about your attitude regarding different statements about climate change. How much do you agree with the following statements?**

1. There is no certain evidence that a long-term warming trend exists.
2. The main reason for the current climate change is human activities. *(inversed, recoded)*
3. Climate change has serious consequences for people and nature. *(inversed, recoded)*
4. Scientists exaggerate the dangers of climate change.
5. strongly disagree
6. somewhat disagree
7. neither agree nor disagree
8. somewhat agree
9. strongly agree
10. not specified

### *Information literacy*

**Next we would like to know how you felt about the reporting on the climate conference in the media which you used. How much do you agree with the following statement?**

The reporting in the media which I used was easy to understand for me.

1. strongly disagree
2. somewhat disagree
3. neither agree nor disagree
4. somewhat agree
5. strongly agree
6. not specified

### *Media use*

**There are many different sources of information available in order to catch up on the latest political and social events. How frequently do you use…**

1. news and information programs on public television, such as Tagesschau, ZDF heute or Weltspiegel (including online media libraries)?
2. news and information programs on private channels, such as RTL Aktuell or Sat1.News (including online media libraries)?
3. a printed national newspaper, such as the Süddeutsche Zeitung or the Frankfurter Allgemeine Zeitung?
4. a printed magazine or a printed weekly newspaper, such as Der Spiegel or Die Zeit?
5. a printed regional daily?
6. BILD-Zeitung (printed)?
7. Spiegel Online (spiegel.de)?
8. BILD Online (bild.de)?
9. other online newspapers (such as sueddeutsche.de, zeit.de)?

(7) several times daily

(6) daily

(5) several times a week

1. once a week

(3) several times a month

1. less often
2. never

**How frequently do you discuss political and social topics with others in personal conversations?**

(7) several times daily

(6) daily

(5) several times a week

(4) once a week

(3) several times a month

(2) less often

(1) never

## *Table e. Descriptive results of independent variables in the first wave*

| **Influencing factors** | **N** | **M** | **SD** | **Min** | **Max** |
| --- | --- | --- | --- | --- | --- |
| ***General individual factors*** | | | | | |
| Age | 1121 | 47.89 | 13.01 | 18 | 69 |
| Gender (1 = female) | 1121 | 0.47 | 0.5 | 0 | 1 |
| Education | 1121 | 3.10 | 1.09 | 1 | 5 |
| Income | 982 | 2.86 | 1.35 | 1 | 7 |
| ***Topic-specific individual factors*** | | | | | |
| Personal importance | 1121 | 3.84 | 1.01 | 1 | 5 |
| Climate change skepticism | 1114 | 2.21 | 0.85 | 1 | 5 |
| Information literacy | 1099 | 3.66 | 0.92 | 1 | 5 |
| Knowledge T1 | 1121 | 2.42 | 1.59 | 0 | 7 |
| ***Sources of information*** | | | | | |
| public TV | 1121 | 4.84 | 1.84 | 1 | 7 |
| commercial TV | 1121 | 4.14 | 1.91 | 1 | 7 |
| national print newspaper | 1121 | 2.15 | 1.51 | 1 | 7 |
| weekly newspaper or magazine | 1121 | 2.12 | 1.28 | 1 | 7 |
| regional print newspaper | 1121 | 3.63 | 2.00 | 1 | 7 |
| tabloid newspaper (BILD) | 1121 | 2.00 | 1.56 | 1 | 7 |
| spiegel.de | 1121 | 2.25 | 1.61 | 1 | 7 |
| bild.de | 1121 | 2.09 | 1.64 | 1 | 7 |
| other online newspaper | 1121 | 2.27 | 1.66 | 1 | 7 |
| interpersonal discussions | 1121 | 4.10 | 1.45 | 1 | 7 |
